# Activation of AMP-Activated Protein Kinase-Sirtuin 1 Pathway Contributes to Salvianolic Acid A-Induced Browning of White Adipose Tissue in High-Fat Diet Fed Male Mice

**DOI:** 10.3389/fphar.2021.614406

**Published:** 2021-05-28

**Authors:** Jianfei Lai, Qianyu Qian, Qinchao Ding, Li Zhou, Ai Fu, Zhongyan Du, Cui Wang, Zhenyuan Song, Songtao Li, Xiaobing Dou

**Affiliations:** ^1^ School of Public Health, Zhejiang Chinese Medical University, Hangzhou, China; ^2^ School of Life Science, Zhejiang Chinese Medical University, Hangzhou, China; ^3^ Molecular Medicine Institute, Zhejiang Chinese Medical University, Hangzhou, China; ^4^ College of Animal Science, Zhejiang University, Hangzhou, China; ^5^ Department of Kinesiology and Nutrition, University of Illinois at Chicago, Chicago, IL, United States

**Keywords:** salvianolic acid A, AMPK, SIRT1, adipocyte browning, obesity

## Abstract

**Background:** Salvianolic acid A (Sal A), a natural polyphenolic compound extracted from *Radix Salvia miltiorrhiza* (Danshen), exhibits exceptional pharmacological activities against cardiovascular diseases. While a few studies have reported anti-obesity properties of Sal A, the underlying mechanisms are largely unknown. Given the prevalence of obesity and promising potential of browning of white adipose tissue to combat obesity, recent research has focused on herbal ingredients that may promote browning and increase energy expenditure.

**Purpose:** The present study was designed to investigate the protective antiobesity mechanisms of Sal A, in part through white adipose browning.

**Methods:** Both high-fat diet (HFD)-induced obese (DIO) male mice model and fully differentiated C3H10T1/2 adipocytes from mouse embryo fibroblasts were employed in this study. Sal A (20 and 40 mg/kg) was administrated to DIO mice by intraperitoneal injection for 13-weeks. Molecular mechanisms mediating effects of Sal A were evaluated.

**Resluts:** Sal A treatment significantly attenuated HFD-induced weight gain and lipid accumulation in epididymal fat pad. Uncoupling protein 1 (UCP-1), a specialized thermogenic protein and marker for white adipocyte browning, was significantly induced by Sal A treatment in both white adipose tissues and cultured adipocytes. Further mechanistic investigations revealed that Sal A robustly reversed HFD-decreased AMP-activated protein kinase (AMPK) phosphorylation and sirtuin 1 (SIRT1) expression in mice. Genetically silencing either AMPK or SIRT1 using siRNA abolished UCP-1 upregulation by Sal A. AMPK silencing significantly blocked Sal A-increased SIRT1 expression, while SIRT1 silencing did not affect Sal A-upregulated phosphorylated-AMPK. These findings indicate that AMPK was involved in Sal A-increased SIRT1.

**Conclusion:** Sal A increases white adipose tissue browning in HFD-fed male mice and in cultured adipocytes. Thus, Sal is a potential natural therapeutic compound for treating and/or preventing obesity.

## Introduction

Obesity is a worldwide public health problem. The chronic energy excess can lead to obesity and further metabolic dysfunctions (Chaput, 2014; Gonzalez-Muniesa et al., 2017). Obesity is characterized by expansion of white adipose tissue and reduced brown adipose tissue activity (Chait and den Hartigh, 2020). While it is difficult in humans to increase brown adipose tissue mass, in recent years, browning of white adipose tissue was identified as a promising tool to reduce obesity (Carpentier et al., 2018). Brown fat tissue dissipates surplus calorie intake into heat energy via a process known as non-shivering thermogenesis (Harms and Seale, 2013; Hanssen et al., 2016). Adaptative thermogenesis occurs mostly in brown fat (Ahmadian et al., 2018), which contains specialized mitochondria-rich brown adipocytes whose thermogenic functionality is conferred by the uncoupling protein 1 (UCP-1) (Pisani et al., 2018; Lettieri-Barbato, 2019). Although brown and white adipocytes originate from different cell lineages and each lineage has a different progenitor, they are proposed to be readily interconvertible to each other (Rosenwald et al., 2013).

Browning or beiging refers to expression of UCP-1 in multilocular cells with thermogenic capability within white adipose tissue. This occurs in presence of some external stimuli, which convert white adipocytes into beige or brite (brown in white) adipocytes (Lim et al., 2012). Ectopic expression of hallmark proteins for brown adipocytes such as UCP-1 in white adipocytes induces acquisition of brown adipose tissue (BAT) features (Tiraby et al., 2003). Cold exposure is a well-established effective way to stimulate adipose tissue thermogenesis. Several pathways have been identified to contribute to cold exposure-induced UCP-1 expression and subsequent WAT browning (Liu et al., 2019), among which, AMP-activated protein kinase (AMPK) (Lee et al., 2020), sirtuin 1 (SIRT1) (Liao et al., 2018), and protein kinase A (PKA) (Cong et al., 2018) stimulate, while p38 mitogen-activated protein kinase (MAPK) inhibits, white adipose tissue browning (Wang et al., 2019). Obesity adversely impacts these signaling pathways, contributing to defective WAT browning in HFD-induced obesity (Zhang et al., 2019).

Searching for safe small molecular compounds that can activate WAT browning is believed to be an effective strategy to improve obesity. A number of dietary compounds and medical herbs have been proposed as tools for increasing energy expenditure and decreasing fat accumulation in mammals (Azhar et al., 2016; Silvester et al., 2019). Danshen, a traditional Chinese medicinal herb, is the dried root and rhizome of *Radix Salvia miltiorrhiza*, and has been widely used for the prevention and treatment of cardiovascular diseases (Cheng, 2007). Salvianolic acid A (Sal A) is one of the main water-soluble phenolic carboxylic acid derivatives in Dansen (Fan et al., 2010). Several studies have reported that Sal A possesses a variety of pharmacological properties, including anti-oxidant, anti-inflammatory, anti-fibrotic and anti-carcinogenic activities (Li et al., 2018; Zhang H. F. et al., 2018; Qin et al., 2019). Previous studies showed that salvianolic acid A intervention effectively reversed obesity induced by HFD (Ding et al., 2016). However, the underlying mechanisms are not fully understood.

In this study, we confirmed that Sal A intervention reversed HFD-induced obesity. We also provided strong evidence that Sal A induced browning in both WAT of HFD-fed mice and in differentiated C3H10T1/2 adipocytes. We demonstrate, for the first time that activation of AMPK-SIRT1 pathway contributed to the browning process via UCP-1 induction. Thus, our study provides new mechanisms by which Sal A exerts antiobesity effects.

## Materials and Methods

### Animals

All experiments described in this study were performed in accordance with the guidelines for animal experiments released by the National Institute of Animal Health. This study is approved by the Animal Ethic Committee of Zhejiang Chinese Medical University. 48 male C57BL/6 mice (8 weeks) had free access to food and water. Mice were housed in a temperature-controlled environment (23 ± 2°C) with a 12 h light/dark cycle. After adapting to the feeding regimen, mice were randomly divided into four groups (*n* = 12), namely, the normal diet (ND) group, high-fat diet (HFD) group (60% fat, D12492, Research Diets, New Brunswick, NJ), HFD with low-dose Sal A intraperitoneal injection (HFD-LS, 20 mg/kg) group, and HFD with high-dose Sal A intraperitoneal injection (HFD-HS, 40 mg/kg) group. Sal A was obtained from Chengdu mansite bio-technology solarbio Co., Ltd. (Sichuan, China). After one-week environmental adaption, mice were fed with normal diet or HFD with or without Sal A intervention for another 13-weeks. Sal A was dissolved in distilled water and administered every other day. Body weight and food intake were measured once per week. At the end of the experiment, all mice were anesthetized with sodium pentobarbital (30 mg/kg body weight) after overnight fasting and sacrificed. Fat tissues were weighed and harvested for further analysis.

### Cell Culture

Mouse embryo fibroblast C3H10T1/2 cells were obtained from American Type Culture Collection (Manassas, VA) and grown in Dulbecco’s modified Eagle’s medium (DMEM, Gino Biomedical Technology Co. LTD., Hangzhou, China) containing 5% fetal bovine serum (FBS, Thermo Fisher Inc., VA), 1.0 μmol/L dexamethasone (Sigma-Aldrich, St. Louis, MO), 10 mg/L insulin (Sigma-Aldrich, St. Louis, MO), and 1.0 μmol/L rosiglitazone (Sigma-Aldrich, St. Louis, MO) for 3 days. Cells were then transferred to DMEM with 5% FBS, 10 mg/L insulin, and 1.0 μmol/L rosiglitazone until 80% of adipocytes differentiated. Cultured medium was re-fed every 2 days. Maturation of adipocytes was confirmed by Oil Red O (Yuanye Biological Technology Co., LTD., Shanghai, China) staining for lipid droplets.

### Histology

The epididymal fat samples were cut into sections (10 μm) using a Leica cryostat and were then stained using hematoxylin and eosin (H&E) to visualize the size of adipocytes in epididymal white adipose tissue (eWAT) using a microscope (OLYMPUS BX51, Japan). The number of cells within four randomly chosen areas (100 × 100 μm) was counted, and the mean value was calculated.

### Ribonucleic Acid Interference

Cultured cells were transfected with mouse siRNA for SIRT1 or AMPK (Santa Cruz, CA) using Lipofectamine 2000 according to the manufacturer’s instructions. In the control group, cells were transfected with scrambled siRNA (Santa Cruz, CA). Gene silencing efficiency was verified by detecting protein content with immunoblotting analysis after transient transfection with siRNA.

### Quantitative-Real-Time Polymerase Chain Reaction

Total RNA was isolated from WAT and cultured adipocytes using RNAiso Plus (Takara, Dalian, China) according to the manufacturer’s instructions, and cDNA was synthesized from RNA using the PrimeScript™ RT Master Mix (Takara, Dalian, China). Quantitative real-time PCR was performed to analyze gene expression using the SYBR Premix ExTap II (Takara, Dalian, China) and the LightCycler 480 System (Roche, Germany). The mRNA levels were normalized to *18s*. Primers for *UCP-1, PGC-1α, Prdm16, Cidea, Fgf21, AMPK,* and *18s* were listed in Supplementary Table S1.

### Western Blot Analysis

Western-blot was performed as previously described (Dou et al., 2018) and the following antibodies were used: anti-phospho-PKA, anti-PKA, anti-phospho-p38, anti-p38, anti-phospho-AMPK, anti-AMPK, anti-phospho-ACC, anti-ACC, anti-SIRT1, anti-UCP-1 and anti-GAPDH (Cell Signaling Technology, Danvers, MA).

### Statistical Analysis

All data are presented as the means ± SD. The statistical analyses were performed using one-way analysis of variance (ANOVA) by SPSS 22.0 software (SPSS Inc., Chicago, IL), followed by the least significant difference (LSD) test for multiple comparisons. *p* < 0.05 was considered statistically significant.

## Results

### Sal a Upregulates Uncoupling Protein 1 Expression in Fully Differentiated C3H10T1/2 Adipocytes

**FIGURE 1:**
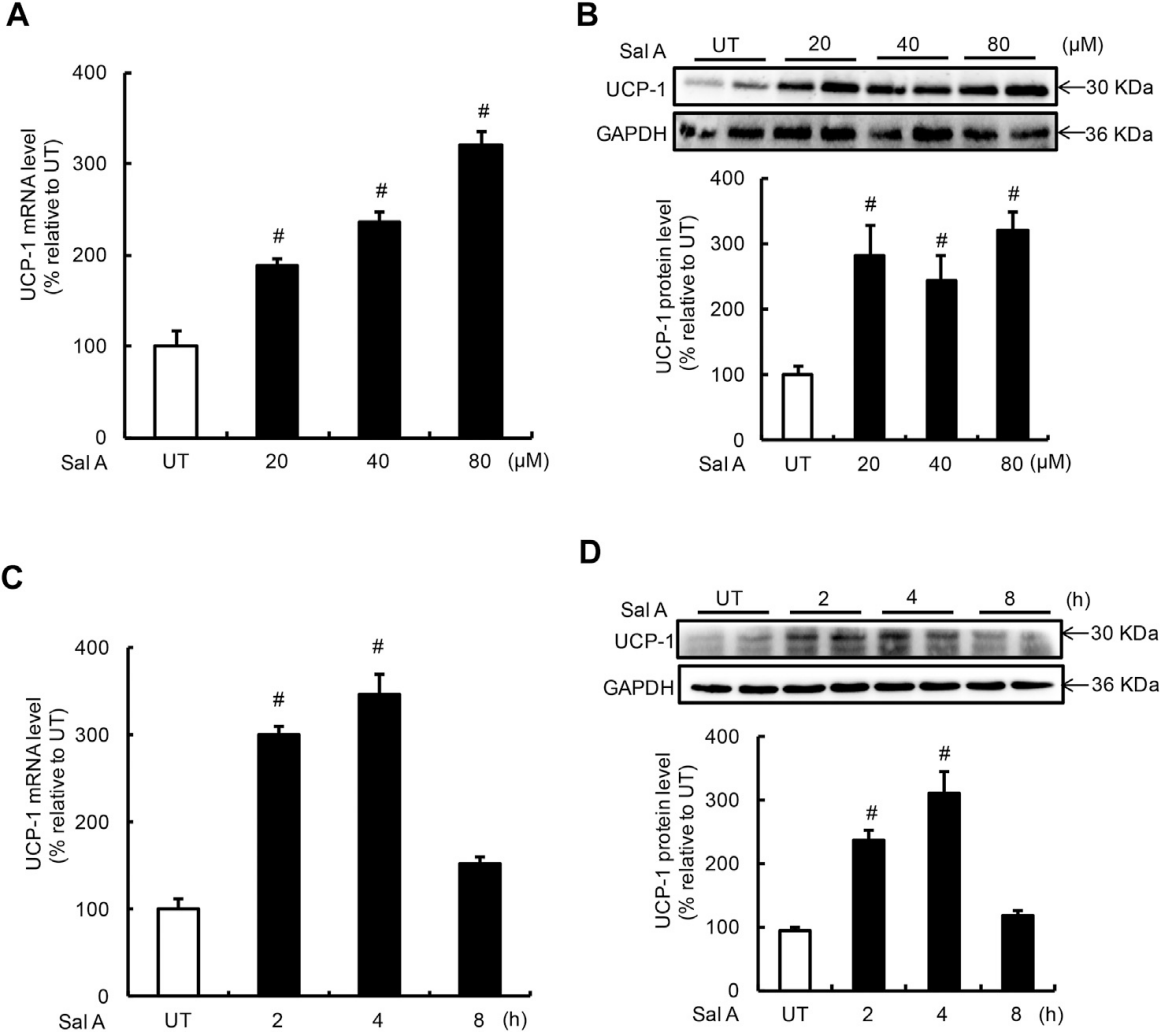
Sal A treatment induced UCP-1 expression in adipocytes. **(A,B)** Differentiated C3H10T1/2 adipocytes were treated with Sal A (0, 20, 40, and 80 μM) for 4 h, intracellular UCP-1 content was determined by RT-PCR and Western blot, respectively. **(C,D)** Time-course changes of intracellular mRNA and protein levels of UCP-1 were determined on Sal A (80 μM) treatment with different duration (0, 2, 4, and 8 h). All values are denoted as means ± SD from three independent batches of cells. #*p* < 0.05 vs. the UT. All groups contain two or three samples (*n* = 2 or *n* = 3).

UCP-1 induction is the predominant feature of white adipocyte browning. The effects of Sal A on UCP-1 expression in adipocytes were investigated by treating fully differentiated C3H10T1/2 adipocytes to exogenous Sal A. Expressions of UCP-1 at both mRNA and protein levels were determined by real time-PCR and Western blotting, respectively. A dose-dependent effect of Sal A on UCP-1 expression was detected when adipocytes were treated with Sal A at 0, 20, 40, and 80 μM, respectively, for 4 h. Both mRNA and protein of UCP-1 were upregulated by Sal A in a dose-dependent manner (Figures 1A,B). Time-course effects of Sal A on UCP-1 expression were subsequently examined using 80 μM exogenous Sal A for 0, 2, 4, and 8 h. As shown in Figures 1A,C,D significant increase of UCP-1 expression at both mRNA and protein levels could be observed at as early as 2 h time point and peaked at 4 h time point. The UCP-1 expression returned to the basal levels at 8 h point.

### AMP-Activated Protein Kinase Activation Contributes to Sal A-Induced Uncoupling Protein 1 Upregulation

**FIGURE 2:**
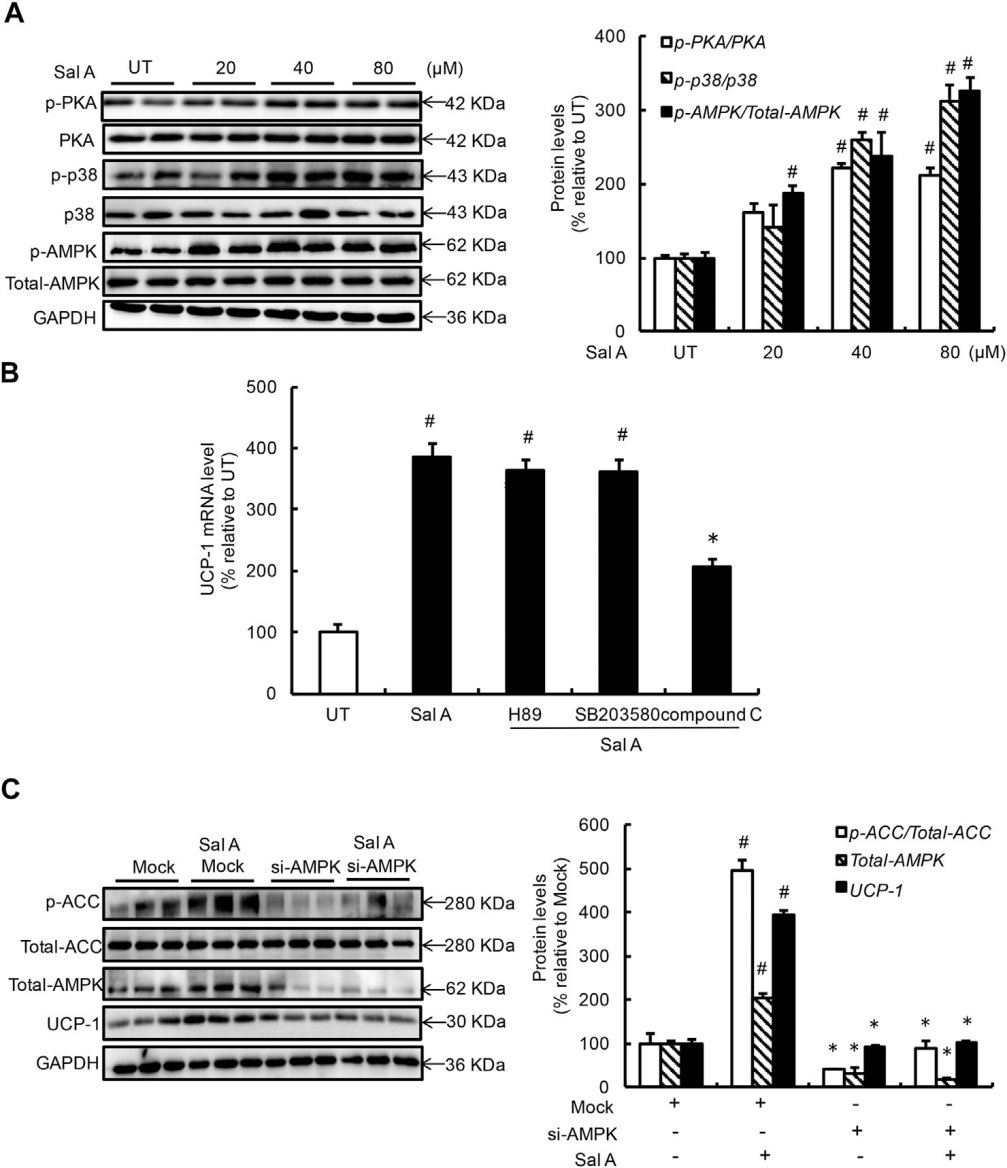
AMPK activation contributes to Sal A-induced UCP-1 upregulation. **(A)** Differentiated C3H10T1/2 adipocytes were treated with Sal A (0, 20, 40, and 80 μM) for 4 h, intracellular *p*-PKA, PKA, p-p38, p38, *p*-AMPK, and AMPK contents were determined by Western blot. **(B)** Fully-differentiated C3H10T1/2 cells were pre-treated with PKA inhibitor (H89, 20 μM), p38 inhibitor (SB203580, 10 μM), and AMPK inhibitor (compound C, 1 μM), respectively, for 2 h before incubated with Sal A (80 μM) for 4 h. Intracellular UCP-1 mRNA level was measured by RT-PCR. **(C)** Fully-differentiated C3H10T1/2 adipocytes were transfected with siRNAs for AMPK. After silencing AMPK by siRNA, cells were exposed to 80 μM Sal A for 4 h. The protein levels of UCP1, AMPK, *p*-ACC and ACC were detected by Western blot. All values are denoted as means ± SD from three independent batches of cells. #*p* < 0.05 vs. the UT or Mock; *p < 0.05 vs. the Sal A treatment group. All groups contain two or three samples (*n* = 2 or *n* = 3).

To delineate the mechanism(s) by which Sal A upregulated UCP-1 expression, we first examined the effects of exogenous Sal A exposure on several signaling pathways/enzymes previously being reported to be involved in the regulation of UCP-1 expression, including PKA, p38, and AMPK. As shown in Figure 2A, a dose-dependent activation of Sal A on all these enzymes was observed after a 4 h Sal A treatment. Subsequent investigations using specific inhibitors, H89 for PKA (20 μM), SB203580 for p38 (10 μM), excluded the implication of PKA and p38 in the observed UCP-1 upregulation in response to Sal A. However, inhibition of AMPK, via either pharmacological (compound C, 1 μM) or genetic (siRNA transfection) approach, blunted Sal A-induced UCP-1 upregulation (Figures 2B,C), suggesting that AMPK activation contributes to Sal A-evoked white adipocyte browning’.

### Sirtuin 1 Activation Contributes to Sal A-Induced AMP-Activated Protein Kinase Activation and Uncoupling Protein 1 Upregulation

**FIGURE 3:**
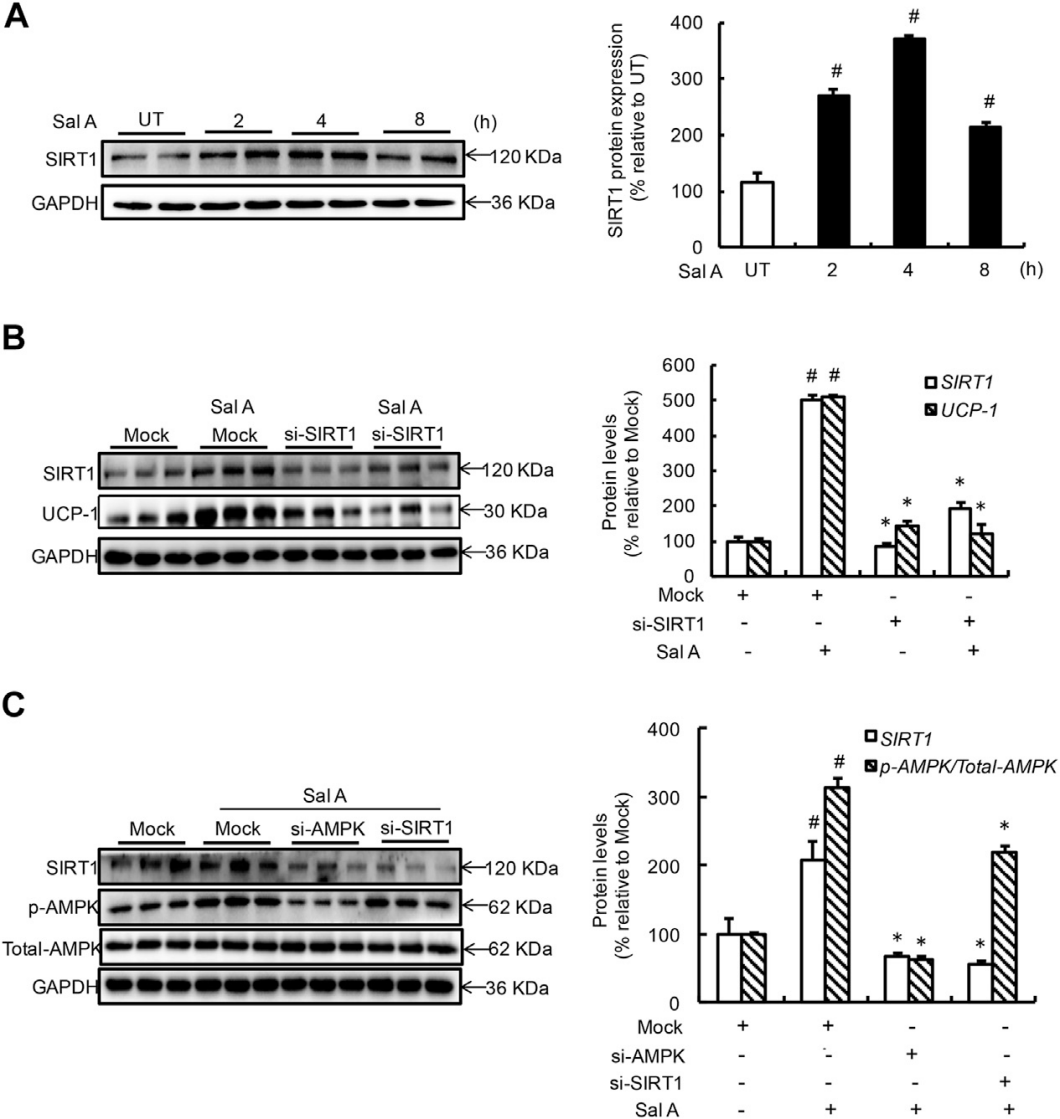
SIRT1 increment underlies Sal A-induced AMPK activation and UCP-1 upregulation. **(A)** Differentiated C3H10T1/2 adipocytes were treated with Sal A (80 μM) for 0, 2, 4 and 8 h. Intracellular SIRT1 contents were determined by Western blot. **(B)** After silencing SIRT1 by siRNA, cells were exposed to 80 μM Sal A for 4 h. The protein levels of UCP-1 and SIRT1 were detected by Western blot. **(C)** C3H10T1/2 adipocytes were transfected with siRNA for SIRT1 or AMPK, respectively. Then cells were treated with Sal A (80 μM) for 4 h. Total cellular lysates were collected for the immunoblotting assay for SIRT1, *p*-AMPK and AMPK. All values are denoted as means ± SD from three independent batches of cells. #*p* < 0.05 vs. the UT or Mock; *p < 0.05 vs. the Sal A treatment group. All groups contain two or three samples (*n* = 2 or *n* = 3).

Both AMPK and SIRT1 activation improved thermogenic program in adipocytes and a mutual regulatory relationship exists between these two enzymes (He et al., 2017; Inagaki et al., 2017). In an attempt to understand whether Sal A also activates SIRT1 and, if so, whether SIRT1 activation mediates Sal A-induced AMPK activation and UCP-1 induction. Time-course changes of SIRT1 activation status were determined by treating fully differentiated C3H10T1/2 adipocytes with 80 mM Sal A for 0, 2, 4, 8 h. As shown in Figure 3A, SIRT1 protein abundance was elevated in response to Sal A exposure and peaked at 4 h time point. To determine if SIRT1 activation is required for Sal A-induced UCP-1 upregulation, we transfected adipocytes with SIRT1 siRNA, followed by exogenous Sal A exposure (80 μM) for 4 h. Importantly, SIRT1 siRNA resulted in an approximate 65% reduction of Sal A-upregulated UCP-1 protein expression (Figure 3B). To further determine the sequence of cellular and molecular signaling events, differentiated C3H10T1/2 adipocytes were transfected with siRNA for either SIRT1 or AMPK, respectively, before been treated with Sal A (80 μM) for 4 h. Whereas AMPK silencing attenuated Sal A-induced SIRT1 upregulation, SIRT1 silencing did not affect AMPK activation by Sal A exposure (Figure 3C), implicating that SIRT1 is a downstream event of AMPK activation in response to Sal A.

### Sal a Supplementation Improves Obesity in High-Fat Diet Fed Mice

**FIGURE 4:**
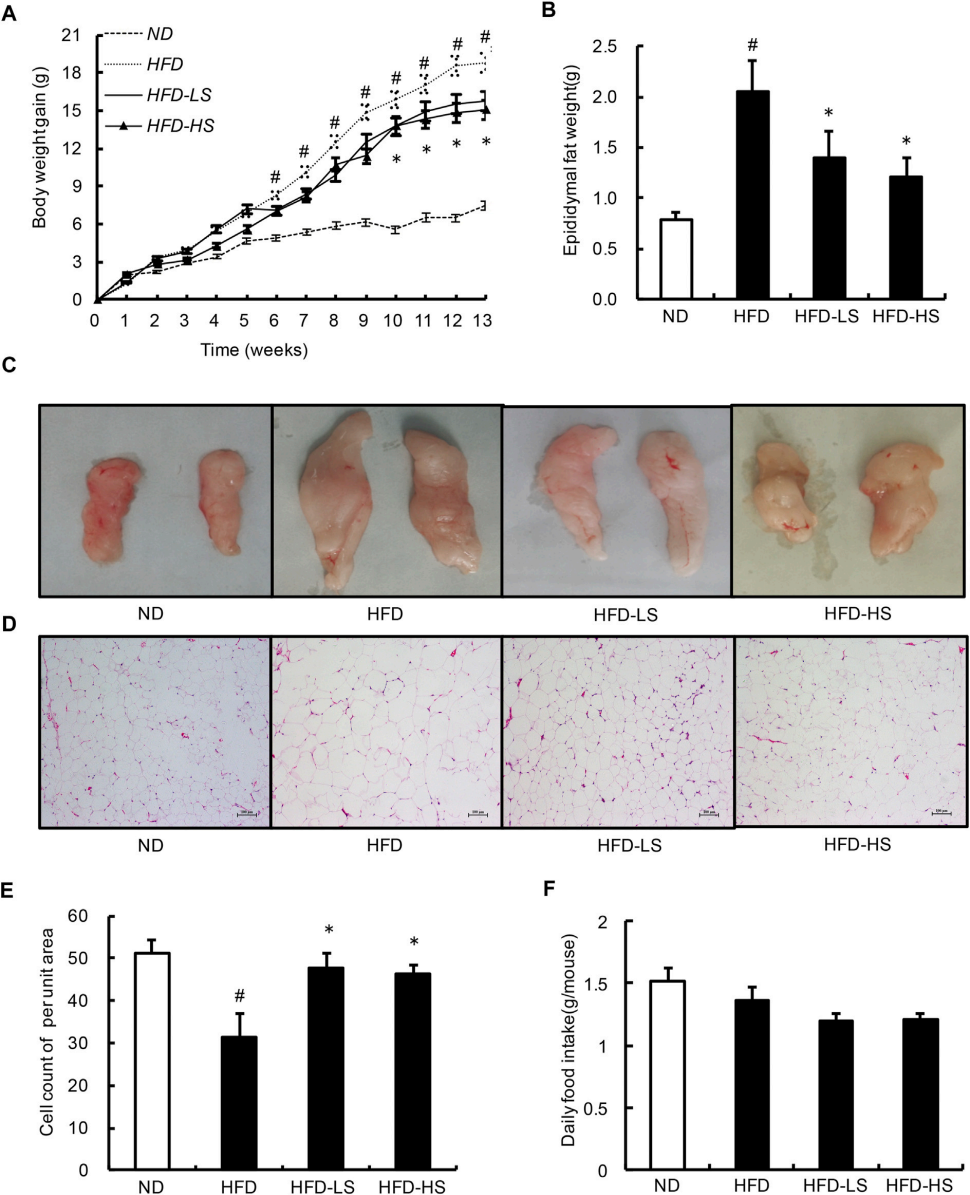
Sal A lowered body weight gain and decreased the eWAT mass and the size of adipocytes within eWAT. **(A)** Change in body weight gain. and **(B)** the eWAT mass. **(C)** Morphological photographs of epididymal fat. **(D)** H&E staining of eWAT to observe the size of adipocytes, scale bar = 100 μm; and **(E)** the cell count of per unit area in H&E-stained sections. **(F)** The daily food intake. # reflects comparing with normal diet (ND) group; * reflects comparing with high-fat diet (HFD) group. All groups contain 12 animals (*n* = 12).

To investigate the *in vivo* relevance of Sal A supplementation as a potential anti-obesity treatment, we fed two doses of Sal A (low dose: 20 mg/kg; high dose: 40 mg/kg) to male C57BL/6 mice (8-week old) on a HFD (60% energy as fat) for 13 weeks. Sal A supplementation, at both doses, ameliorated HFD-induced body weight gain (Figure 4A), which was associated with significantly lowered adiposity (Figures 4B,C), eWAT adipocyte hypotrophy (Figure 4D), and hyperplasia (Figure 4E) in Sal A-treated mice compared to HFD-fed mice. We did not observe any statistical differences between the two doses of Sal A for the above markers. Also, there was no difference in food intake between the HFD and Sal A-treated groups (Figure 4F).

### Sal a Supplementation Improves AMP-Activated Protein Kinase-Sirtuin 1 Pathway Activation and Prevents Uncoupling Protein 1 Downregulation in Epididymal White Adipose Tissue of High-Fat Diet-Fed Mice

**FIGURE 5:**
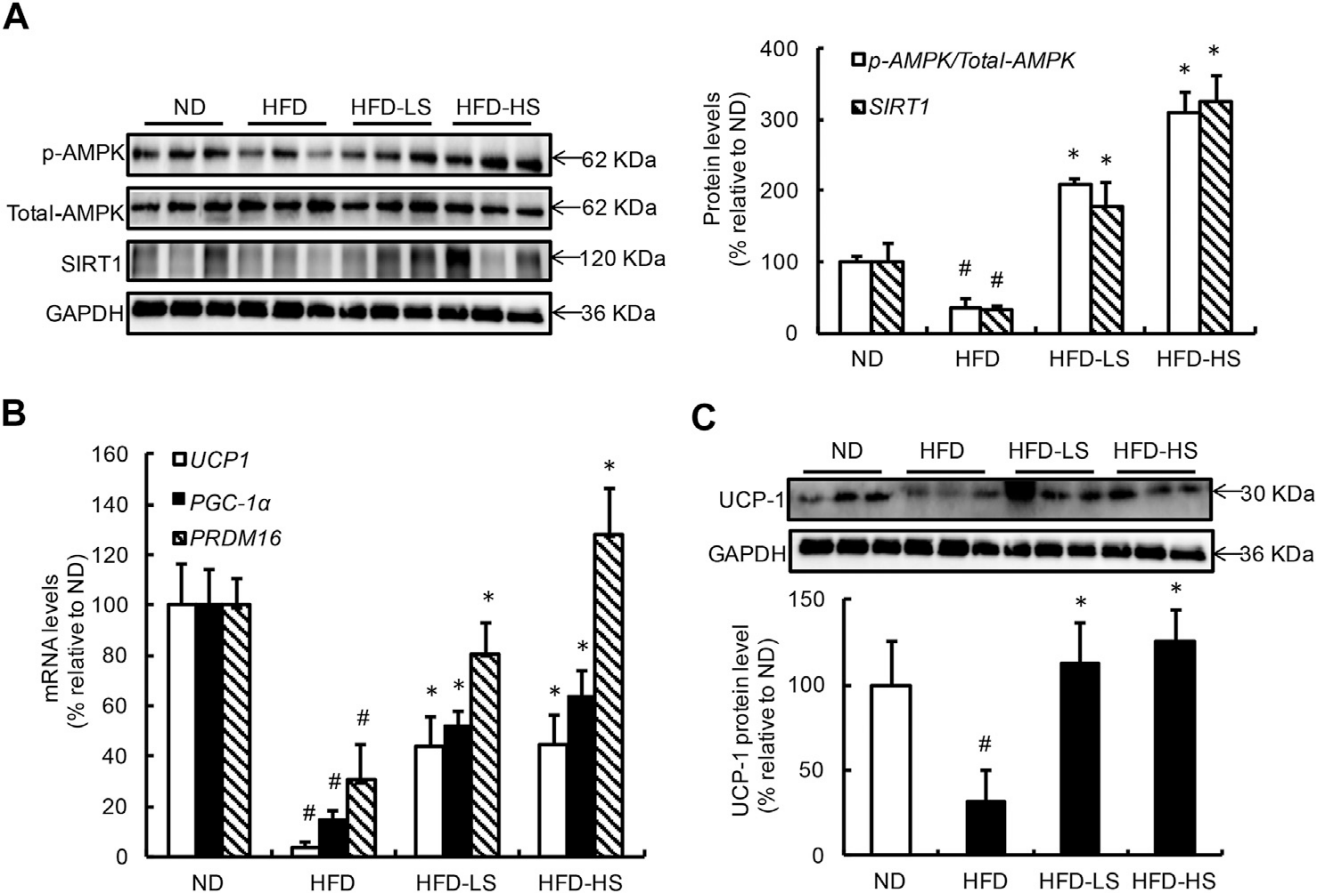
Sal A supplementation improves AMPK-SIRT1 pathway activation and prevents UCP-1 downregulation in eWAT of HFD-fed mice. **(A)** Results of the Western blot analysis of AMPK signaling in eWAT, including *p*-AMPK, and AMPK, SIRT1. **(B)** The gene expressions of UCP1, PGC-1α, and Prdm16, related to WAT browning. **(C)** The protein levels of UCP1 and quantification of the bands. The same internal reference (GAPHD) was used for *p*-AMPK, AMPK, SIRT1, and UCP1 quantitative analysis, since the results come from the same experiment based on the same batch of loading samples. In order to satisfy the logical rationality of the argument, we put the results in panel **(A,C)**, respectively. #*p* < 0.05 vs. the ND group; *p < 0.05 vs. the HFD group. All groups contain 12 animals (*n* = 12).

The effect of Sal A supplementation on AMPK-SIRT1 pathway activation as well as UCP-1 expression in eWAT from different groups were subsequently measured. HFD feeding resulted in AMPK inhibition in eWAT (Figure 5A). However, when compared with the HFD group, the *p*-AMPK/AMPK ratio was increased by approximately 5.8- and 8.7-fold in the HFD-LS and HFD-HS groups, respectively (Figure 5A). HFD feeding decreased Sirt1 expression in eWAT, which was rescued by Sal A supplementation (Figure 5A). We also analyzed the mRNA expression of thermogenic genes, including *Cidea*, *Fgf21*, and *AMPK* in eWAT. Our data showed that Sal A intervention significantly reversed HFD-caused reduction of these genes (Supplementary Figure S1A). Moreover, HFD led to a 96% reduction of UCP-1 mRNA when compared with control mice (Figure 5B). Both doses of Sal A supplementation rescued UCP-1 mRNA reduction in eWAT of HFD-fed mice (Figure 5B). The protein expression of UCP-1 in both subcutaneous (inguinal) and brown adipose tissue were also detected in our study. Sal A intervention did not improve HFD-reduced UCP-1 in subcutaneous (inguinal) adipose tissue (Supplementary Figure S1B). In brown adipose tissue, there was no significant difference in UCP-1 expression among the groups (Supplementary Figure S1C). To strengthen our conclusion, we measured gene expression of PGC-1α and Prdm16, two critical genes controlling mitochondrial biogenesis and adipose tissue browning, using adipose tissues obtained previously. As shown in Figure 5B, both genes show similar changes as UCP-1 does. The protein expression of UCP-1 in eWAT of HFD-fed mice was notably lower than that in the ND-fed mice, which were reversed by Sal A supplementation at both doses (Figure 5C).

## Discussion

The present study documents for the first time that Sal A, a natural polyphenolic compound extracted from *Radix Salvia miltiorrhiza* (Danshen), protects against obesity in a long-term high-fat diet (HFD) feeding mouse model through activating white adipose tissue (WAT) browning process. Our mechanistic investigations further reveal that Sal A induces WAT browning via eliciting the AMPK-SIRT1 pathway activation in adipocytes.

Obesity and related comorbidities are major health concerns. Individuals with obesity have a substantially higher risk of developing many diseases, such as type 2 diabetes mellitus, hyperlipidemia, and cardiovascular diseases (Rangel-Huerta et al., 2019). Thus, the search for compounds that have potential to prevent or even reverse obesity development has been intensified. Sal A is an important bioactive water-soluble ingredient in Danshen, which is widely used in functional foods and drugs in China for the treatment and prevention of cardiovascular diseases (Wang et al., 2017). The positive association between obesity and cardiometabolic health has been well-established (Kachur et al., 2017), however, the potential anti-obesogenic role of Sal A has received little investigative attention. Obesity occurs as a combined result of WAT expansion and compromised adipose tissue (white and beige adipose) browning (references), which otherwise can protect against obesity via increasing energy expenditure by generating heat (Rui, 2017). To the best of our knowledge, the current study is the first research to investigate the potential anti-obesity function of Sal A and elucidate underlying mechanisms.

Using long-term HFD feeding mouse model of obesity in male C57BL/6 mice and clinically relevant doses of Sal A (Salvianolic acid A) (Mu et al., 2020), our results showed that Sal A treatment markedly reversed HFD-induced body weight gain, which was associated with a significantly reduced HFD-induced the mass of eWAT gain. The morphological observations showed that the HFD-fed mice with Sal A administration had smaller-sized adipocytes within eWAT when compared to these in HFD-fed mice. The browning of WAT provides a new perspective in the identification of therapeutic strategies for weight loss. Several studies have focused on the relationship between the anti-obesity effects of plant extracts and WAT browning (Wang et al., 2015; Lone et al., 2016). UCP-1, a thermogenic protein, is abundantly expressed in BAT. The increase of UCP1 expression in WAT induces the formation of beige adipocytes (Roh et al., 2018). In this study, we observed that Sal A administration significantly increased mRNA and protein abundance of UCP-1 in both eWAT of mice fed with HFD and cultured adipocytes, indicative of enhanced WAT browning. Due to the lack of metabolic monitoring equipment, we were not able to measure energy metabolism in these mice, which is a limitation of our study.

The underlying mechanism(s) by which Sal A stimulated UCP-1 upregulation remains unknown. The cAMP/PKA pathway plays a central role in inducing UCP-1 expression and adipose tissue browning (Meng et al., 2017). It has been reported that Sal A activated PKA in human umbilical artery smooth muscle cells (Sun et al., 2016). These reports spurred us to explore whether PKA activation is attributable to Sal A-triggered UCP-1 upregulation in adipocytes. In line with our conjecture, Sal A treatment enhanced PKA phosphorylation in cultured adipocytes, however, H89, a special chemical inhibitor of PKA, failed to block Sal A-stimulated UCP-1 expression, suggesting that PKA is not involved in Sal A-promoted WAT browning. Given that genetic ablation of p38 in adipose tissues facilitated WAT browning upon cold stress and prevented diet-induced obesity (Zhang S. et al., 2018) and Sal A inhibited p38 MAPK signal pathway in variety of cells (Zhang et al., 2017; Zhang H. F. et al., 2018; Feng et al., 2020), we subsequently analyzed the possibility of p38-mediated UCP-1 induction in Sal A-treated adipocytes. Unexpectedly, Sal A exposure indeed enhanced p38 activation in cultured adipocytes. Our observation is consistent with a previous report which showed Sal A supplementation stimulated p38 phosphorylation in the brain tissue of subarachnoid hemorrhaged rat (Gu et al., 2017), suggesting that Sal A regulates p38 signaling pathway in a cell type- and/or tissue-specific manner. Furthermore, our data that SB203580, a specific p38 inhibitor, failed to block Sal A-stimulated transcriptional activation of UCP-1, excluded the involvement of p38 in Sal A-promoted WAT browning.

AMPK is a well-recognized energy sensor, which plays an important role in the regulation of cellular energy homeostasis (Zhang et al., 2009). AMPK activation promoted thermogenesis in both brown and WAT (Zhang et al., 2014; Wu et al., 2018), whereas AMPK ablation resulted in cold intolerance and a reduction in non-shivering thermogenesis in mouse adipocytes (Desjardins and Steinberg, 2018). To this end, we subsequently analyzed the involvement of AMPK in Sal A-provoked UCP-1 up-regulation. Our results clearly indicated that Sal A intervention markedly enhanced AMPK phosphorylation in both WAT of HFD-fed mice and fully-differentiated C3H10T1/2 adipocytes. In support of our finding, several lines of evidence reported that Sal A supplementation stimulated AMPK in hepatic and skeletal muscle cells and sciatic nerve (Yu et al., 2012; Qiang et al., 2015). Importantly, compound C, a commonly used chemical inhibitor of AMPK, effectively suppressed Sal A-stimulated UCP-1 increase, indicating that AMPK is required for the induction of browning in WAT by Sal A.

SIRT1 is an intranuclearly located NAD^+^-dependent deacetylase and plays an important role in the regulation of WAT browning (Qiang et al., 2012). UCP-1 in WAT was dramatically decreased in SIRT1^−/−^ mice, while increased in SIRT1 overexpressed mice in response to cold (Qiang et al., 2012). Several studies documented that Sal A supplementation up-regulated SIRT1 in the liver of experimental animals (Xu et al., 2013; Zhu et al., 2018). Based on these observations, we examined whether SIRT1 could potentially contributes to Sal A-promoted UCP-1 upregulation in WAT in mice and our data demonstrated that SIRT1 expression was enhanced by Sal A in both WAT of HFD-fed mice and in fully-differentiated C3H10T1/2 adipocytes, and was involved in Sal A-induced UCP-1. Previous studies have reported the mutual regulatory role between AMPK and SIRT1 in different experimental settings (He et al., 2017; Thirupathi and de Souza, 2017). In this study, we investigated the cross-talk between AMPK and SIRT1 under Sal A treatment. Our data showed that AMPK silencing significantly blocked Sal A-increased SIRT1 expression, while SIRT1 silencing did not affect Sal A-upregulated phosphorylated-AMPK, indicating AMPK was involved in Sal A-increased SIRT1. A well-established mechanism accounting for AMPK-induced SIRT1 activation is the upregulation of Nampt, a rate-limiting enzyme for intracellular NAD + biosynthesis via the salvage pathway, leading to cellular NAD + elevation. It has also been reported that NAD + -enhancing agents including nicotinamide mononucleotide (NMN) not only activate SIRT1 activity but also upregulate its expression, implying that AMPK activation, via upregulating Nampt expression and resultant cellular NAD + elevation, is capable of increasing Sirt1 expression (Song et al., 2019).

The potential mechanisms linking Sal A-regulated AMPK activation are still unclear. Commonly, AMPK is stimulated by two classical signals. One is Ca^2+^-dependent pathway, which is mediated by calcium/calmodulin-dependent protein kinase kinase β (CaMKKβ), and the other one is AMP-dependent pathway, which is regulated by liver kinase B1 (LKB1) (Qiang et al., 2015; Lin and Hardie, 2018). Although how Sal A-regulated AMPK activation in adipocytes is still unclear, recent evidence confirmed that CaMKKβ inhibitor could significantly block Sal A-activated AMPK in HepG2 cells implying that a Ca^2+^-dependent pathway may contribute to Sal A-induced AMPK activation (Qiang et al., 2015).

In conclusion, the present study provides evidence that Sal A administration is protective against HFD-induced obesity in mice. Sal A administration promotes WAT browning in mice fed with HFD, evidenced by an increased UCP-1 expression in WAT of long-term HFD-fed mice with Sal A administration. Mechanistically, we uncovered that the AMPK-SIRT1 pathway activation contributes to Sal A-induced UCP-1 upregulation in adipocytes. Our results suggest that Sal A represent a promising therapeutic choice for the prevention and/or treatment of obesity, as well as its metabolic complications. The future clinical studies are warranted.

## Data Availability

The raw data supporting the conclusions of this article will be made available by the authors, without undue reservation, to any qualified researcher.

## Abbreviations

AMPK, AMP-activated protein kinase; BAT, brown adipose tissue; CaMKKβ, calcium/calmodulin-dependent protein kinase kinase β; CREB, cAMP-response element binding protein; Fgf21, Fibroblast Growth Factor 21; HFD-HS, HFD with high-dose Sal A intraperitoneal injection; HFD-LS, HFD with low-dose Sal A intraperitoneal injection; HFD, high-fat diet; LKB1, liver kinase B1; ND, normal diet; MAPK, p38 mitogen-activated protein kinase; PKA, protein kinase A; PGC-1α, peroxlsome proliferator-activated receptor-γ coactlvator-1α; Prdm16, PR Domain Containing Protein 16; Cidea, Recombinant Cell Death Inducing DFFA Like Effector A; SIRT1, sirtuin 1; UCP-1, uncoupling protein 1; WAT, white adipose tissue.
