# Supplementary material for: Activation of AMP-Activated Protein Kinase-Sirtuin 1 Pathway Contributes to Salvianolic Acid A-Induced Browning of White Adipose Tissue in High-Fat Diet Fed Male Mice
Source: Front Pharmacol. 2021 May 28;12:614406. doi: 10.3389/fphar.2021.614406 (PMC8193940; doi:10.3389/fphar.2021.614406)
Supplement: Supplementary file 1 [file Table1.DOCX]

Table 1. List of primers.

| Gene | Forward primer (5’-3’) | Reverse primer (5’-3’) |
| --- | --- | --- |
| *AMPK* | AGGTGGACATCTGGAGCA | GGCTGATTACTGAAGGGT |
| *Cidea* | TTAAGAGACGCGGCTTTGGG | GAAACTCGAAAAGGGCGAGC |
| *Fgf21* | CCAGTTTGGGGGTCAAGTCC | ACTTTCTGGACTGCGGTGTG |
| *PGC-1α* | TATGGAGTGACATAGAGTGTGCT | GTCGCTACACCACTTCAATCC |
| *Prdm16* | CTTAGCCGGGAAGTCACAGG | CATTGCATATGCCTCCGGGT |
| *18s* | GGGAGGTAGTGACGAAAAAT | ACCAACAAAATAGAACCGCG |
