# Supplementary figures and images for: Activation of AMP-Activated Protein Kinase-Sirtuin 1 Pathway Contributes to Salvianolic Acid A-Induced Browning of White Adipose Tissue in High-Fat Diet Fed Male Mice
Source: Front Pharmacol. 2021 May 28;12:614406. doi: 10.3389/fphar.2021.614406 (PMC8193940; doi:10.3389/fphar.2021.614406)

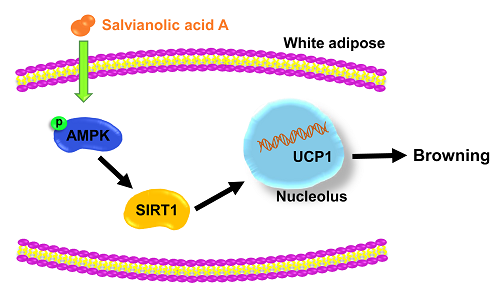

Supplement: Supplementary file 2 [file Image2.tif]
