# Supplementary material for: Activation of AMP-Activated Protein Kinase-Sirtuin 1 Pathway Contributes to Salvianolic Acid A-Induced Browning of White Adipose Tissue in High-Fat Diet Fed Male Mice
Source: Front Pharmacol. 2021 May 28;12:614406. doi: 10.3389/fphar.2021.614406 (PMC8193940; doi:10.3389/fphar.2021.614406)
Supplement: Supplementary file 3 [file Image1.pdf]

Supplementary data

Figure S1

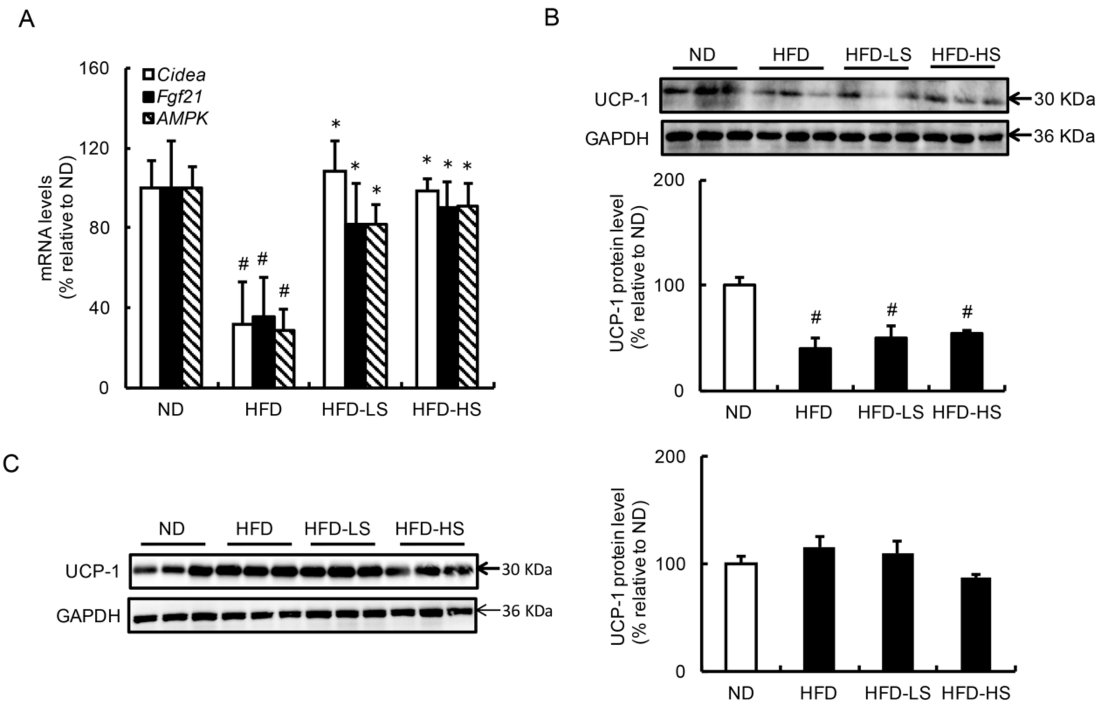


Figure S1. Sal A supplementation improves eWAT browning but not effect UCP-1 in subcutaneous (inguinal) and brown adipose tissues of HFD-fed mice. (A) The gene expressions of *Cidea, Fgf21, and AMPK* in eWAT. (B) The expression of UCP-1 in subcutaneous (inguinal) adipose tissues was detected by Western blot. (C) Protein expression of UCP-1 in brown adipose tissues. # $p < 0.05$ versus the ND group; * $p < 0.05$ versus the HFD group. All groups contain 12 animals (n = 12).
